# Direct-seq: programmed gRNA scaffold for streamlined scRNA-seq in CRISPR screen

**DOI:** 10.1186/s13059-020-02044-w

**Published:** 2020-06-08

**Authors:** Qingkai Song, Ke Ni, Min Liu, Yini Li, Lixia Wang, Yingying Wang, Yingzheng Liu, Zhenxing Yu, Yinyao Qi, Zhike Lu, Lijia Ma

**Affiliations:** 1 School of Life Sciences, Westlake University, Hangzhou, 310024 China; 2 Institute of Biology, Westlake Institute for Advanced Study, Hangzhou, 310024 China

## Abstract

CRISPR-based genome perturbation provides a new avenue to conveniently change DNA sequences, transcription, and epigenetic modifications in genetic screens. However, it remains challenging to assay the complex molecular readouts after perturbation at high resolution and at scale. By introducing an A/G mixed capture sequence into the gRNA scaffold, we demonstrate that gRNA transcripts could be directly reverse transcribed by poly (dT) primer together with the endogenous mRNA, followed by high-content molecular phenotyping in scRNA-seq (Direct-seq). With this method, the CRISPR perturbation and its transcriptional readouts can be profiled together in a streamlined workflow.

## Introduction

Systematic gene perturbation by clustered regularly interspaced short palindromic repeats (CRISPR) is a technological breakthrough for understanding gene function in high-throughput. The wide application of this system is emphasized by the easy access to reagents, scalability, as well as the flexibility of modifying the system to fit versatile application scenario. To further expand the applications, modifications to the intrinsic CRISPR system have been developed. One category is using new or evolved endonuclease with distinct functions, for example, dead Cas9 (dCas9) [1], Cpf1 [2], and Cas13a [3]. And the dCas9 is often fused with other effector proteins including transcriptional activator or repressor and DNA modifier, by which the editing ability of the CRISPR system is extended from changing the DNA sequence to transcriptional and epigenetic regulations [4–6]. Another category is focusing on programing the scaffold RNA. Structural biological evidence has proved that the tetraloop, loop2, and the tail at the secondary structure of the guide RNA transcript do not directly interact with the endonuclease and therefore provide room for the addition of other sequences [7]. The RNA aptamer inserted into the guide RNA provides additional function expansion. Transcriptional effectors or fluorescence molecules are fused with the cognate RNA-binding partners which are further recruited to the RNA scaffold and then targeting sequences [8, 9]. The combinatorial use of the two categories, modifying the endonuclease and programming the scaffold RNA, offers a varied tool kit for the entire CRISPR system covering DNA editing, epigenetic modifying, transcriptional regulation, and genome imaging.

One of the biggest demanding of the CRISPR editing is that how to profile the transcriptome together with genotype at high resolution, for example, in the CRISPR screens. The linkage between the genotype and phenotype is usually established by quantifying the enriched or depleted guide RNAs from cells with the selected phenotypes. In order to add the transcriptome profile as another layer of information, four groups of pioneers combined the single-cell RNA-seq (scRNA-seq) with the CRISPR screening assay, from which the gRNA indexes were captured and barcoded together with the endogenous mRNA via oligo (dT) [10–13]. These methods aimed to generate polymerase II (Pol II)-transcribed gRNA copy or gRNA-associated barcode, which will be poly-adenylated and then captured together with endogenous mRNA during the reverse transcription (RT), so that the genotype, transcriptome, and phenotypes could be all linked at the single-cell resolution. However, most approaches involved complicated cloning strategy and sometime comprised by the decoupling of gRNA spacer and its barcode. CROP-seq generated a poly-adenylated gRNA copy during virus integration and did not suffer from the decoupling; however, the cassette size limited its application with multiplexed gRNAs [12]. Recently, the 10x Genomics Inc. released a single-cell 3′ RNA-seq kit serving the same purpose, which was reported in a recent publication [14]. Differently, they designed two capture sequences and directly incorporated them into the gRNA scaffold, so that the polymerase III (Pol III)-transcribed gRNAs will carry these specific sequences and can be captured by the complementary primers that have been engineered into the 10x GEM beads [14]. However, these “capture sequence” relies on special RT primers and makes the design incompatible with other single-cell RNA-seq platforms, but only the 10x platform.

In this study, we developed Direct-seq, a framework to combine scRNA-seq with CRISPR screen by employing a novel programmed gRNA scaffold, which can serve as index gRNA without incorporating other RT primers. We show that a mixed adenosine/guanine sequence can be inserted into three different locations of the scaffold with no influence on the performance of the CRISPR/Cas9 and CRISPRa systems. Meanwhile, this mixed A/G sequence could be efficiently captured with the endogenous mRNA via poly (dT) on different single-cell RNA-seq platforms. This study exhibits a highly flexible and easy-to-access framework to index gRNAs across versatile CRISPR applications, so that the gene expression profile after genome perturbation can be identified together with the genotype at single-cell resolution.

## Results

### Programmable RNA scaffold with capture sequences

**Fig. 1:**
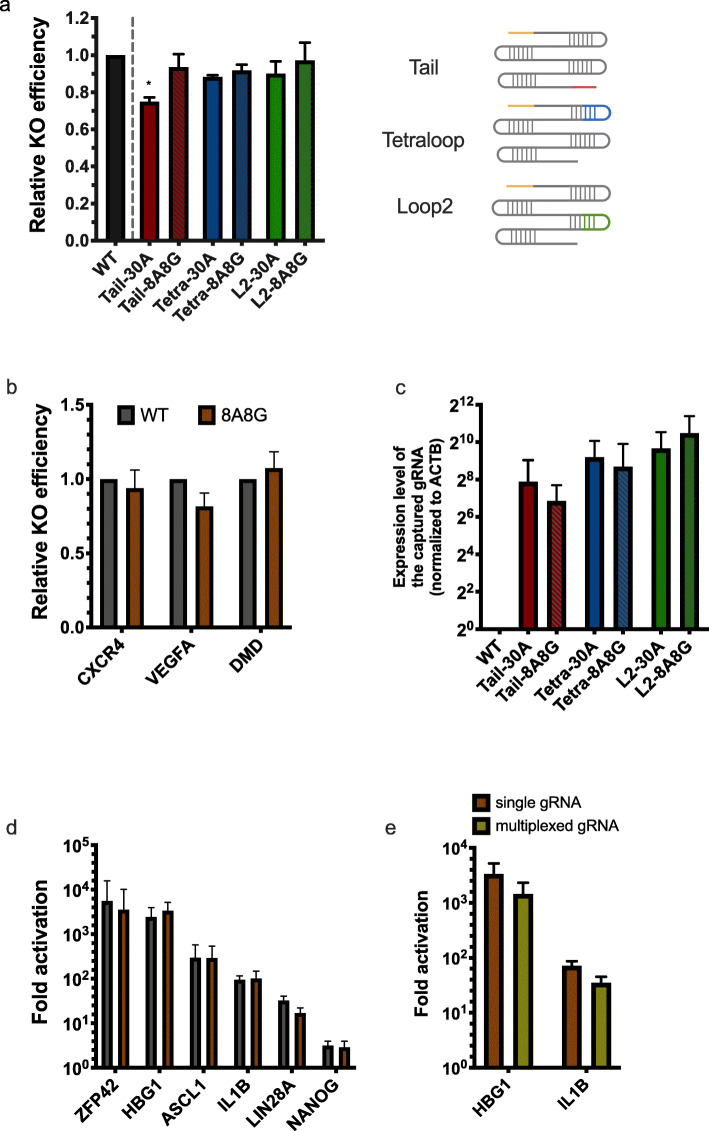
The editing efficiency of the programmed scaffold. **a** The relative CRISPR KO efficiency was estimated using spacer sequence targeting human EMX1 gene. The KO efficiency of variant scaffolds was all normalized to the WT scaffold. “Tail”, tail position in the scaffold. “Tetra,” tetraloop position in the scaffold. “L2,” loop2 position in the scaffold. “30A,” capture sequence with 30 consecutive adenosine. “8A8G,” a 30-nt length capture sequence composes eight leading A and mixed with G every seven continuous A (AAAAAAAAGAAAAAAAGAAAAAAAGAAAAA) (*P ≤ 0.05, compared with WT in unpaired *T* test). **b** The CRISPR KO efficiencies of three spacers targeting CXCR4, VEGFA, and DMD were estimated. The “8A8G” capture sequence was inserted to the Tail position on each of these scaffolds. **c** The capture efficiencies of the gRNA transcripts were estimated using RT-qPCR. In all of the Tail, Tetra, and L2 positions, both 30A variant and 8A8G variant scaffolds were examined. In each position, the capture efficiencies of the 30A and 8A8G variant scaffold were normalized to the WT scaffold. **d** The CRISPR activation assay was conducted on different targeting genes, and expression increase was estimated using RT-qPCR. **e** The activation efficiencies of the single gRNA and the multiplexed two gRNAs were compared. The brown bar indicated the fold activation when single gRNA was used, and the light green bar indicated the fold activation when a co-expressed (HBG1 gRNA and IL1B gRNA) cassette was used. The scaffold used here was the same as **d**

In order to enable direct capture of the gRNA transcript with poly (dT) RT primers, we firstly explored the possibility of introducing 30 consecutive adenosines (30A) into the gRNA scaffold. Presumably, this 30A sequence will be part of the gRNA transcript and captured by poly (dT) primer in RT. We inserted the 30A into three locations of the scaffold, tetraloop (Tetra), loop2 (L2), and tail (Tail), inspired by the crystal structure of Cas9/gRNA complex and other previous studies [7, 8, 14–16]. We transfected the wild-type gRNA (WT) and the three programmed gRNAs (Tail-30A, Tetra-30A, and L2-30A) into the 293T cells and compared their editing efficiency using the online tool TIDE [17] (the “Methods” section). The results showed that the editing efficiency dropped 24% with the 30A insertion at the tail position, whereas 13% and 12% with the insertion at the tetraloop and loop2 respectively (Fig. 1a and Additional file 2: Table S1). This analysis indicated that the direct insertion of poly(A) sequences into the gRNA scaffold negatively impacted the editing efficiency, especially at the tail position.

We then looked for alternative capture sequences that could minimize or eliminate the influence on the editing efficiency, while remain the capturability by poly (dT) primer. We started the optimization at the tail position since the addition of sequence at this position is less likely to interfere the gRNA folding and more flexible in different applications. In some scenarios, the tetraloop and loop2 are often occupied by RNA aptamers for recruiting other effectors. For example, the SAM system [8], the CRISPRainbow [9], and the CRISPR-Display [18] all fall into this category. We suspected that the long poly(A) stretch might influence the CRISPR editing efficiency since the poly(A)-binding protein (PABP) may obstruct the proper formation of the Cas9/gRNA complex [7]. We reasoned that an ideal capture sequence could be a poly(A) stretch mixed with certain frequency of other nucleotide, in which the consecutive A ensured the efficient base pairing with poly (dT) primer, while the addition of non-A nucleotide might prevent the interfering from PABP. Deo and colleagues reported that the minimal length of poly(A) stretch for effective PABP binding is 11 nt, which sets the maximum length of consecutive adenosine in the capture sequence [19]. Meanwhile, studies also show that the G-T pairing is the most stable non-canonical base pairing [20], and guanylated tails is the most frequent mixed-tail in multiple species [21]. Inspired by these previous works, we mimicked the guanine addition into the poly(A) sequence and replaced the original 30A with an 8A8G insertion, in which one guanine was incorporated with every seven adenosines except the first eight leading adenosine (Additional file 3: Table S2). With the new 8A8G tail insertion (Tail-8A8G), the editing efficiency recovered to 94% relative to the WT (Fig. 1a and Additional file 2: Table S1). We then incorporated the “8A8G” capture sequence into the tetraloop (Tetra-8A8G) and loop2 (L2-8A8G) positions and noticed that this A/G mixed capture sequence also recovered the editing efficiency as it works at the tail position (Fig. 1a and Additional file 2: Table S1). We would like to mention that extending the stem length of loop2 could further increase the editing efficiency slightly, which might due to the extended stems contributed to stabilize the stem-loop structure than the original stems. For example, at the position of loop2, the stem extension worked best when we extended the stem from 4 to 10 bp, with the Tm increased to 40 °C, which is higher than the temperature of 37 °C in most in vitro or in vivo conditions (Additional file 1: Figure S1 and Additional file 3: Table S2).

In order to test whether the modified scaffold works well with other targeting sites, we conducted the same analysis on three other targets selected from literatures (CXCR4, VEGFA, and DMD) by using the Tail-8A8G scaffold. The relative editing efficiencies to WT were at similar level as the EMX1, but varied across targeting sites (CXCR4 93.8%, VEGFA 81.6%, and DMD 107.3%) (Fig. 1b and Additional file 2: Table S1). Meanwhile, multiple known off-targeting sites were examined for VEGFA. These sites have been reported with varied off-targeting rates [22]. The results showed that the addition of the A/G mixed capture sequences did not increase the off-targeting rate, regardless of the insertion position, across all sites we checked (Additional file 1: Figure S2).

Next, we investigated the capture efficiency of the A/G mixed sequence in reverse transcription by RT-qPCR (Fig. 1c, the “Methods” section). The results suggested that the 8A8G scaffold could be captured efficiently, and the efficiencies are at similar level to that of the “30A” scaffold.

In summary, these results suggested that the use of 8A8G programmed scaffold could enable the direct capture of the gRNA transcript using poly (dT) in reverse transcription, while retaining the knockout efficiency of the CRISPR/Cas9 genome editing.

### The programmed scaffold is compatible with CRISPRa and multiplexed perturbation

Next, we tested whether the programmed scaffold is compatible with other types of CRISPR perturbations. As a demonstration, we employed the CRISPRa SAM system, in which both tetraloop and loop2 were occupied by the MS2 aptamer [8]. Firstly, we tested how well the expression of targeting gene can be elevated after applying CRISPR activation with the programmed scaffold. The capture sequence was cloned to the tail position, which had been demonstrated working well in the CRISPR KO system (Additional file 3: Table S2). Similar to the KO assay, our results indicated that the Tail-8A8G could promote the gene expression to a comparable activation level as the wild type SAM system on different targeting sites (Fig. 1d). Considering the potential application of the programmed scaffold in combinatorial perturbation, we also examined the performance of the 8A8G scaffold when activating two tandem gRNAs. We specifically tested this application because the previous barcode-based methods were not able to characterize the identities of multiplexed gRNAs in transcriptome profiling [14, 23]. As shown in Fig. 1e, we identified similar level of activation from two multiplexed gRNAs expressed from the same cassette (Fig. 1e, Additional file 3: Table S2). Together, these two cases suggested that the 8A8G programmed scaffold was compatible with other CRISPR systems like CRISPRa, as well as multiplexed perturbation.

### The programmed scaffold could be used as index gRNA in different single-cell RNA-seq platforms

To establish a method for versatile platforms, we explored the compatibility of the programmed scaffold with the Chromium 10x 3′ and 5′ single-cell RNA-seq kits (Additional file 1: Figure S3, a and b), and the Fluidigm C1 platform applying the Takara SMART-seq v4 protocol (Additional file 1: Figure S3, c). These platforms covered wide range of single-cell RNA-seq methodologies serving different throughputs.

For the droplet-based platforms like 10x 3′ and 5′ single-cell RNA-seq, transcripts from one droplet share the same cell barcode (CBC). Therefore, the gRNA transcripts can be enriched from the pre-amplified cDNA and sequenced as an index gRNA library, separated from the mRNA library. The index gRNAs and the endogenous mRNA from the same cells can be combined by the CBC in data analysis afterwards. Given this rationale, the 5′ kit is readily compatible with our programmed scaffold. The spacer region, which serves as the perturbation index, is sitting between the 10x barcode oligo and the invariable region of the scaffold, so that it can be directly enriched from the pre-amplified cDNA (Additional file 1: Figure S3b).

**Fig. 2:**
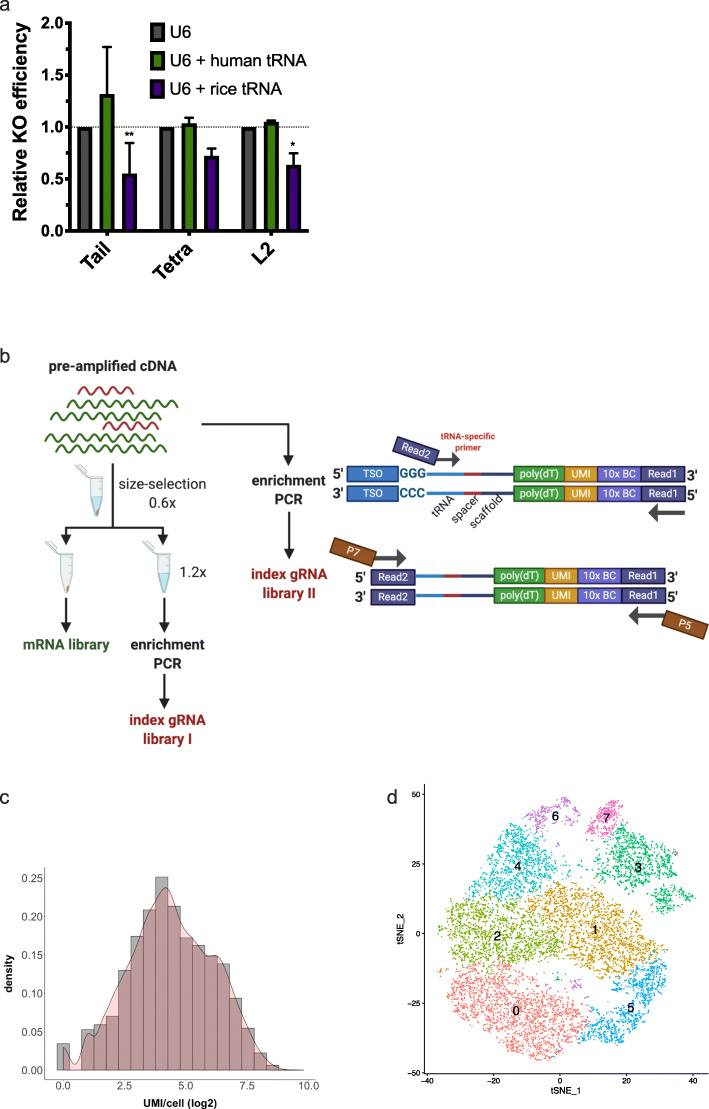
The demonstration of the programmed scaffold in single-cell CRISPR screen. **a** Two tRNA sequences from human (Gln) and rice (Gly) were incorporated into the gRNA expression cassette, locating at the downstream of the U6 promoter. The editing efficiencies were examined for gRNA scaffolds with the capture sequence inserted at the tail, tetraloop, and loop2 (*P ≤ 0.05, **P ≤ 0.01, compared with U6 in 2-way ANOVA test followed by Tukey’s multiple comparisons test). **b** Workflow of the library preparation using the 10x 3′ single-cell RNA-seq kit. Two index gRNA libraries were generated from the pre-amplified cDNA via nested PCR. Half of the cDNA was directly used as the PCR template; the other half was size selected as described in the 10x protocol. The 1st PCR of the nested PCR used a tRNA-specific primer to enrich gRNA-derived cDNA. The 2nd PCR added the P5 and P7 to enable NGS sequencing. The mRNA library was prepared as stated in the 10x protocol. **c** Histogram of the gRNA UMI per cell from the index gRNA library. **d** The tSNE cell clustering of the 10x data

However, the application with the 10x 3′ kit requires some modification to the original protocol, since there is no primer binding site for the same purpose. We explored a solution by simply including a tRNA sequence into the gRNA expression cassette. Indeed, the combined use of tRNA and gRNA has been reported in many previous studies to either boost the gRNA expression level or process multiplexed gRNAs from the same transcript [24–28]. To work with the 10x 3′ kit and serve as primer binding region, the tRNA should locate at the downstream of the U6 promoter and upstream of the gRNA sequence. To do so, we firstly tested the editing efficiency of the programmed scaffold with tRNA incorporated (Fig. 2a). The results suggested that the human Gln tRNA retained editing efficiency, no matter where the 8A8G capture sequence was inserted. To also take advantage of the tRNA characteristics that can process multiplexed gRNAs from the same transcript, we designed a dual-gRNA expression cassette with the tRNA sequence sitting between the two gRNAs and generated a CRISPR library to demonstrate the application of the 8A8G programmed scaffold using the 10x 3′ kit (Fig. 2b). Since previous barcode-based methodologies for single-cell CRISPR screen are restricted from delivering of multiplexed gRNA, we sought to use this dual-gRNA single-cell CRISPR screen to demonstrate the expanded utility of our method, besides the compatibility and scalability.

We collected the post-screening cells and conducted the single-cell RNA-seq using the 10x 3′ v3 kit (Fig. 2b, the “Methods” section). The gRNA transcripts embedded with the 8A8G capture sequence were captured by the poly (dT) alongside with the adenylated endogenous transcripts. As shown in Fig. 2b, the pre-amplified cDNA was divided into two halves. One half was size selected following the 10x protocol; the bead elution was used to make the mRNA library, and the supernatant was used to make the index gRNA library (I). The other half was used for making another index gRNA library (II) directly. We made these two index gRNA libraries to examine whether the size selection is beneficial to enrich the interested molecules or is actually causing material loss (the “Methods” section, Additional file 4: Table S3). We used nested PCR to specifically enrich the cDNA derived from gRNA transcripts, then added sequencing adapters (Fig. 2b, Additional file 1: Figure S4). Although only cDNA from transcripts that had not been fully processed into two separated gRNAs (containing tRNA sequence) could be enriched by the nested PCR, the sequencing data indicated that this strategy recovered 99.4% cell barcodes (CBC) identified from the mRNA library (13,350 from library I and 13,355 from library II, out of 13,435 from the mRNA library) (Additional file 5: Table S4, the “Methods” section). Since the data also show excellent correlation between the two index gRNA libraries (Additional file 1: Figure S5), we recommend the user to just use the index gRNA library I, which is in accordance with the standard protocol.

Out of the 35 million sequencing reads from the gRNA index library, 81.1% was mapped back to the reference, while only 37% of which from the 10x 3′ kit using CS2 was successfully aligned [14] (Additional file 5: Table S4, the “Methods” section). This might due to non-specific CS2 RT products that were libraried together with the gRNA-transcribed cDNA. The number of UMI varied across cell barcodes (Fig. 2c, Additional file 1: Figure S6), which is consistent with the index gRNA libraries prepared with the 10x 3′ kit [14]. And the median UMI per cell is 20 given ~ 2000 sequencing reads per cell. Furthermore, in order to use the “high-content molecular phenotyping” readout from the scRNA-seq experiment, we analyzed and clustered the cells according to the transcriptome profiles from the mRNA library (Fig. 2d, Additional file 1: Figure S7). Although the input cells were relatively less heterogeneity since they were collected as the bottom population of CD69 negative cells from cell sorter, they still exhibited very differently when examined the transcriptome in high resolution, which demonstrated the necessity of combinatorial use of the scRNA-seq with CRISPR screen.

Besides the 10x 3′ scRNA-seq experiment, we also conducted a small-scale demonstration using the Fluidigm C1 system, which represented relatively low throughput application and characterized the cellular transcriptome individually rather than relying on cell barcodes. As a proof-of-principle, we collected cells from a screen experiment, in which K562-Cas9 cells were infected by a lenti-CRISPR library in MOI 0.3. Among all 73 cells we investigated with the Fluidigm C1 microfluidic chip, gRNA transcripts were successfully characterized in 93.2% (68) cells (Additional file 1: Figure S8 and Additional file 5: Table S4, the “Methods” section). Since scaffold specific primer was not incorporated to enrich the gRNA-derived cDNA, the sequencing depths for detecting index gRNA were relatively higher compared to the depth per cell in droplet-based experiment (Additional file 5: Table S4). Therefore, a nested PCR that was similar to what have been applied in the 10x 3′ demonstration is highly recommended for higher detection sensitivity (Additional file 1: Figure S3, a and c).

Taken together, these data demonstrated the compatibility of the A/G mixed capture sequence on multiple single-cell RNA-seq platforms. By mimicking the adenylated endogenous mRNA, gRNA transcripts could be directly captured by poly (dT) primer during the reverse transcription and serve as perturbation index in high identification rate.

## Discussion

In this study, we developed "Direct-seq", a framework of direct “genotyping” for single-cell CRISPR screen, by incorporating a capture sequence into the gRNA scaffold. With that, genotype, transcriptome, and phenotype can be analyzed simultaneously. We used an A/G mixed capture sequence to mimic the poly(A) tail of pol II transcripts and facilitate the direct annealing of gRNA with poly (dT) RT primer that is widely used in versatile scRNA-seq platforms.

We demonstrated almost 100% identification rate of the index gRNA, when using the 10x 3′ scRNA-seq kit given ~ 2000 sequencing reads per cell. Although the 10x kits (v3 and above) offer gRNA indexing through two specifically designed capture sequences, our methods exhibited similar or even better performance in terms of the enrichment of gRNA and the identification rate of index gRNA across cells [14]. The better enrichment was reflected from both the fragment analysis after the three sequential steps of amplifications (Additional file 1: Figure S4) and higher alignment rate of the sequencing reads (Additional file 5: Table S4). We speculate the performance of our method is benefited from the nested PCR we applied, which employed a gRNA specific primer (Fig. 2c and Additional file 1: Figure S3a), while the primers used in the 10x protocol may not exclude the non-specific products that have annealed with the CS1 and CS2 RT primers. We would like to note that the incorporation of tRNA is required to apply the 8A8G programmed scaffold with the 10x 3′ kit. Indeed, the combined use of tRNA and gRNA has been applied in many previous studies [24–28], and the editing efficiency was retained well in our tests (Fig. 2a). Besides the 10x 3′ kit, our programmed scaffold is also compatible with the 10x 5′ kit, and we would recommend using a gRNA scaffold-specific primer to enrich the molecules of interests for better performance (Additional file 1: Figure S3, b). We also demonstrated the application of the programmed scaffold on the Fluidigm C1 platform following the SMART-seq protocol and identified gRNA from 93.2% of cells we have tested. However, without the enrichment step, higher sequencing depths per cell would be required to identify the gRNA reads from endogenous transcriptome (Additional file 5: Table S4). Importantly, among the applications across versatile platforms, our methodology does not require modifying the original protocols until the cDNA has been pre-amplified. This not only expands the compatibility of our method with different scRNA-seq assays, but also eliminates the potential disturbing to the critical reverse transcription step. For example, additional RT primers may competitively anneal with poly-adenylated RNA molecules and disturb the transcriptome profiles.

Under the rationale of the 8A8G programmed scaffold, some other capture sequences were also examined, including the addition of the G nucleotide at different frequency and including additional tractor sequences to the Tail-8A8G to see whether they changed the gRNA structure stability and influenced the editing efficiency [14] (Additional file 1: Figure S9). Although we did not comprehensively examine these sequences, some of them exhibited promising performance at least from the perspective of editing efficiency and may potentially be used as alternative capture sequences in gRNA scaffold for direct poly (dT) annealing and single-cell CRISPR screen.

There are three lines of solutions to enable scRNA-seq with gRNA index after CRISPR screen. One is to include a gRNA barcode into the gRNA expression vector, which provided a poly-adenylated barcode transcript but suffered from decoupling of gRNA and its barcode at single-cell level [10, 11, 13, 29]. On the other hand, CROP-seq has been designed to include a gRNA cassette within 3′ LTR, which is duplicated during viral integration, and the entire expression cassette will then be expressed as a poly III transcript for genome editing and a pol II transcript for scRNA-seq [12]. However, as the gRNA cassette has to be designed sitting within 3′ LRT, the cassette size needs to be limited, and therefore, the compatibility with multiplexed gRNA genome editing and screening is low. Recently, the 10x Genomics offers commercial solution by incorporating additional capture sequence into gRNA, which enables gRNA capture by specifically designed RT primers [14]. As we have discussed in the previous sections, this commercialized method relies on the engineered GEM beads of the 10x kit and could be affected by the compatibility with other scRNA-seq platforms. Together, here we presented a novel programmed scaffold with an A/G mixed capture sequence, which enables streamlined and scalable single-cell CRISPR screen on varied scRNA-seq platforms and in versatile applications.

## Methods

### Plasmid design and construction

The gRNA expression vectors were constructed by assembling the scaffold variants with a modified lentiGuide-puro backbone (Addgene #52963). The Goldengate assembly reaction was set as the following: 30 cycles of 5 min at 37 °C and 5 min at 22 °C, followed by a 30-min heat inactivation at 65 °C. Clones were propagated in Stbl3 chemically competent cells. And plasmids were extracted using QIAprep SpinMiniprep kit (QIAGEN #27106) and verified by Sanger sequencing. All the scaffold variants used in this study were summarized in the Additional file 3: Table S2. And primers and spacer sequences are showed in Additional file 4: Table S3.

### Cell line establishment and cell culture

K562 wildtype was purchased from American Type Culture Collection (ATCC). Jurkat wildtype was a gift from Xu Li Lab, Westlake University. HEK293T wildtype was a gift from Shang Cai Lab, Westlake University. HEK293T wildtype was grown in high-glucose DMEM (SIGMA #D6429) with 10% fetal bovine serum (FBS) (Gemini #900-108) and 1% penicillin/streptomycin (Gibco #15140-122). K562 and Jurkat wildtype were grown in RPMI 1640 (SIGMA #R8758) with 10% FBS (Gemini #900-108) and 1% penicillin/streptomycin (Gibco #15140-122). Stable cell lines were established to streamline the perturbation experiments. In brief, a HEK293T-Cas9 cell line was established by inserting the lentiCas9-Blast (Addgene #52962) lentivirus into the wild type HEK293T cells. Monoclone was selected, expanded, and maintained with 2 μg/mL blasticidin. The Jurkat-Cas9 and K562-Cas9 cell line used in the CRISPR screen experiments were established following the same procedure. In the case of CRISPR activation, a HEK293T-dCas9-MPH-VPR cell line was established by inserting the lentiMPHv2 (Addgene #89308) and lenti-dCAS-VP64 Blast (Addgene #61425) lentivirus into the wild type HEK293T cell. Monoclone was selected, expanded, and maintained with both blasticidin (2 μg/mL) and hygromycin antibiotics (200 μg/mL). The Cas9 and dCas9 expression were confirmed by western blot (mouse anti-Cas9, 7A9-3A3, Cell Signaling).

The HEK293T-Cas9 and HEK293T-dCas9-MPH-VPR cells were cultured in high-glucose DMEM (SIGMA #D6429) supplemented with 2 mM GlutaMax (Gibco #35050-061), 1 mM sodium pyruvate (Gibco #11360-070), 10% heat-inactivated characterized FBS (GEMINI #900-108), and 1% penicillin/streptomycin (Gibco #15140-122).

The Jurkat-Cas9 and K562-Cas9 cells were cultured in RPMI-1640 (SIGMA #R8758) supplemented with 10% heat-inactivated characterized FBS (GEMINI #900-108) and 1% penicillin/streptomycin (Gibco #15140-122).

All cell lines were subject to periodic testing for mycoplasma using MycoBlueTM Mycoplasma Detector (Vazyme D101-02).

### CRISPR perturbation and editing efficiency estimation

Cells were plated at the density of 3 × 10^5^ per well in 2 mL media in poly-D-lysine-coated 6-well plates. After 24 h, cells typically reached 90% confluency, and then 1 μg plasmids were transfected with Lipofectamine 3000 (Invitrogen #L3000-015) according to the manufacturer’s instructions.

For gene knockout experiments, 1.5 × 10^5^ mKate2-positive cells were sorted by fluorescence-activated cell sorting (FACS) 72 h post-transfection. The sorted cells were lysed, and genomic DNA was extracted using TIANamp Genomic DNA Kit (TIANGEN #DP304-03). The targeting regions were PCR amplified using the NEBNext High-Fidelity 2X PCR Master Mix (NEB #M0541S) according to the manual. Sanger sequences of the targeting PCR products were analyzed with the TIDE webtool (http://tide.nki.nl). Default parameters were used for the analysis. The indel size range was set as 10–35 bp. The bar plots in the figures of this manuscript all exhibited the mean ± s.e.m. from 2~4 replicates, unless otherwise indicated.

For gene activation experiments, 3 × 10^5^ mKate2-positive cells were sorted by fluorescence-activated cell sorting (FACS) 72 h post-transfection. Cells were lysed, and RNA was extracted using the miRNeasy Mini Kit (Qiagen #217004). cDNA was synthesized using the PrimeScript RT reagent Kit with gDNA Eraser (TAKARA #RR047A) using 200 ng of RNA per cDNA reaction. For quantitative PCR, the reactions were prepared using the SYBR Green PCR Master Mix (Life Technologies #4309155) with 1 μL cDNA per reaction in a 20-μL reaction volume. The expression level of GOI was normalized to *ACTB*. Cycling conditions were set as the following: 98 °C for 30 s, 98 °C for 10 s, 66 °C for 30 s, and 72 °C for 10s. The latter three steps cycled for 40 times with plate reads taken after the 72 °C step. For bulk cell RNA-seq, cDNA product from 100 ng RNA was firstly purified using the QIAquick Nucleotide Removal Kit (QIAGEN #28306). And 1 ng purified product was used for library preparation by the TruePrep DNA Library Prep Kit V2 for Illumina (Vazyme #TD503), according to the manufacturer’s instruction. The final libraries were enriched by a 0.4–0.9× double-sided selection with AMPure XP beads and eluted in 25 μL of nuclease-free H_2_O.

For capture experiments, the detection method was the same as the gene activation experiment except the reverse transcription primer (Additional file 4: Table S3).

### Screen library preparation for 10 × 3′ scRNA-seq demonstration

A dual-sgRNA library plasmid (12,472 sgRNA pairs), pMD2.G (Addgene#12259) envelope plasmid, and psPAX2 (Addgene#12260) packaging plasmid were transfected with calcium chloride into HEK293T cells at 80% confluency. Lentiviral supernatant was collected at 48 h and 72 h post-transfection, filtered through a 0.45-μm filter, and then concentrated by ultra-centrifuging at 70,000*g*, 4 °C for 2 h, using Beckman Optima with SW28 rotor and Ultra-clear tube (Beckman #344058). Finally, the concentrated lentivirus was aliquoted and stored at − 80 °C until use.

A total of 20 × 10^6^ target cells were infected at MOI ≤ 0.3 in RPMI-1640 containing 8 μg/mL polybrene by centrifuging at 700*g*, 32 °C for 2 h. At 48 h post-transduction, cellular mKate2 expression, indicating the successful transduction, was tested by flow cytometry (Cytoflex, Beckman). Then, the 10 days of puromycin selection started. In the period of puromycin selection, culture medium with puromycin and blasticidin at the concentration of 2 μg/mL was added every 2 days, and the cell concentration in culture medium was maintained at 5 × 10^5^/mL. Meanwhile, cellular mKate2 expression was monitored by flow cytometry until the ratio of mKate2+ cells was higher than 95%, which indicated the end of puromycin selection.

A total of 20 × 10^6^ Jurkat cells were stimulated by ImmunoCult™ Human CD3/CD28 T Cell Activator (STEMCELL) in RPMI with 10%FBS at the concentration of 5 × 10^6^/mL. The activator was added at the dose of 25 μL/mL. After 24 h of stimulation, cells were harvested and stained for surface CD69 (FN50, Biolegend), and 2 × 10^6^ CD69- (Bottom 25%) cell population was sorted using FACS (Fusion, BD) for single-cell sequencing.

### scRNA-seq on the 10 × 3′ RNA-seq platform

Sequencing libraries were prepared using the Chromium Single-Cell 3′ Reagent Kits v3 (PN-1000075), Chromium Single-Cell B Chip kit (PN-1000153), and Chromium i7 Multiplex Kit (PN-120262). The Chromium Single-Cell 3′ Reagent Kits v3 User Guide (10x Genomics, CG000184) protocol was followed until the cDNA was pre-amplified (the 1st PCR).

After that, cDNA was divided into two 40 μL aliquots. One aliquot was size-selected following the 10x protocol: the 0.6x left-sided size selection products were used to make the mRNA library, and 0.6–1.2× double-sided size selection products (eluted in 25 μL) were used to make the index gRNA library I. The other aliquot was used for making another index gRNA library (II), right after a 1.2x AMPure bead purification (eluted in 25 μL).

In order to make the index gRNA libraries, nested PCR was employed to enrich the gRNA amplicons and then incorporate the sequencing adapters.

1. For each sample, a total of eight gRNA enrichment PCR (the 2nd PCR) reactions were conducted, with each reaction including 3 μL template, 25 μL NEBNext® Ultra™ II Q5® Master Mix (NEB #M0544S), 2.5 μL tRNA_Read2 primer (10uM), 2.5 μL P5_read1 primer (10uM), and nuclease-free water up to 50 μL (Additional file 4: Table S3). The PCR program was set as the following: (1) 98 °C for 30 s; (2) 14 cycles of 98 °C for 10 s, 60 °C for 10 s, then 72 for 10 s; and (3) 72 °C for 2 min. The PCR products from each sample were combined and purified by 0.7–1.0× double-sided size selection and eluted in 80 μL nuclease-free water.
2. For each sample, a total of five library preparation PCR (the 3rd PCR) reactions were performed. Each reaction included 10 μL purified products from the 2nd PCR, 25 μL NEBNext® Ultra™ II Q5® Master Mix, 2.5 μL P7_Read2_Index1 (or index2) primer (10uM), 2.5 μL P5_read1 primer (10uM), and nuclease-free water up to 50 μL (Additional file 4: Table S3). The PCR program was set as the following: (1) 98 °C for 30 s; (2) 5 cycles of 98 °C for 10 s, 54 °C for 15 s, then 65 °C for 20 s; and (3) 72 °C for 2 min. Finally, the resulting index gRNA libraries were cleaned up via a 0.7–1.0× AMPure XP double-sided size selection and sent for sequencing.

The mRNA library was prepared following to the Chromium User Guide. A detailed protocol has been uplodaed to Protocol Exchange (http://dx.doi.org/10.21203/rs.3.pex-952/v1).

### scRNA-seq on the Fluidigm C1 platform

A sgRNA screen library was prepared and transduced into the K562-Cas9 cell. A small amount of cells was collected to demonstrate the application on the C1 platform.

The single-cell sequencing libraries were prepared using the Fluidigm C1 Single-Cell Auto Prep System, with C1 Single-Cell Auto Prep IFC for mRNA seq (Fluidigm #100–5760). The cell was prepared following the C1 protocol. The cDNA collected from the microfluidic chip was purified via QIAquick Nucleotide Removal Kit (QIAGEN# 28306). For each individual cell, 1 ng purified product was used for library preparation via the TruePrep DNA Library Prep Kit V2 for Illumina (Vazyme #TD503). The final libraries were enriched by a 0.7–1.5× double-sided selection with AMPure XP beads and eluted in 25 μL nuclease-free H_2_O.

### Analysis of droplet-based scRNA-seq data

The 10x scRNA-seq data was processed using the Cell Ranger Single-Cell Software (v.3.0.1). The sequencing reads of the mRNA library were aligned to the human genome (hg38) with default parameters, and reads from the index gRNA libraries were aligned to their own references. The processed data from the gRNA libraries and the mRNA library were combined according to the 10x cell barcode. If a mRNA cell barcode was also identified from the index gRNA libraries, the cell was considered as indexed by the gRNA.

To perform cell clustering, Seurat (v.3.1.1) was applied to further remove low-quality cells and run tSNE analysis. Cells with more than 7000 or less than 200 detected genes, as well as those with mitochondrial transcripts proportion higher than 35%, were excluded. The top 10 principal components (PCs) were used to do clustering (with a resolution of 0.4) by t-stochastic neighbor embedding (tSNE) with the default settings of the RunTSNE function.

The published 10 × 3′ and 5′ scRNA-seq data were downloaded from GEO GSE146194 and processed in the same way.

The enrichment analyses were conducted by R package clusterProfiler (v.3.8.1). The enrichment score was calculated from gRNA targeting genes within a cluster against all targeting genes in the library, and the top10 KEGG categories were used in the plot which shows the major functional categories.

### Analysis of C1 scRNA-seq data

The gRNA scaffold was identified from the Read1 of fastq files. We calculated the read count per gRNA across all cells, and HTseq (v.0.11.2) was used to calculate the reads count for each gene.

## Supplementary information

**Additional file 1: Figure S1**. An illustration shows the stem extension when incorporating longer loop in order to main stable secondary structure. **Figure S2**. The off-target rate was examined for six known off-targeting sites of the VEGFA gRNA. **Figure S3**. Workflows of applying the programmed scaffold in different single cell RNA-seq platforms. **Figure S4**. The products from gRNA transcripts were effectively enriched by the nested PCR as exhibited from the fragment size analysis. **Figure S5**. The pearson correlation of UMI per cell between the two index gRNA libraries. **Figure S6**. Histogram of the gRNA UMI per cell from the index gRNA library II. **Figure S7**. Transcriptome profiling and KEGG enrichment of gRNA targeting genes in single cell sequencing. **Figure S8**. The sequencing reads from gRNA transcripts in each single cell were estimated using single cell RNA-seq conducted on the Fluidigm C1 platform. **Figure S9**. Different scaffold variants were examined, including G addition at different frequencies and A/G mixed sequence with different tractors.

**Additional file 2: Table S1**. Comparison results of KO efficiency used WT and progammed scaffold RNA in gene knockout experiment.

**Additional file 3: Table S2**. List of scaffold variants, tRNA, and sgRNA cassette. (a) Programmed scaffold variants used in this study. (b) tRNA sequences for gene knockout experiments. (c) Dual-sgRNA expression cassette for Jurkat TCR activation experiment. (d) Design for multiplexed gRNAs expression cassette in the activation experiment.

**Additional file 4: Table S3**. List of primers and spacer sequences. (a) Primers and spacer sequences for gene knockout and activation experiments. (b) Primers and targeting sequences for off-target detection. (c) Primers for capture experiment. (d) Primers used in the nested PCR of gRNA index library preparation.

**Additional file 5: Table S4**. Summary of the sequencing data. (a) Data stats of sequencing after data analysis in this study. (b) Next Generation Sequencing Analysis of the single-cell Transcriptomes of the K562-Cas9 cell infected with lentiviral sgRNA library containing programmed scaffold RNA.

**Additional file 6.** Review history.

## Data Availability

The accession number for the raw sequencing data reported in this paper is NCBI Sequence Read Archive (SRA): GSE143880 [30] and GSE148820 [31]. The scripts used in this study has been deposited in Github: https://github.com/LijiaMALab/scCRISPR [32]
