# Supplementary material for: Direct-seq: programmed gRNA scaffold for streamlined scRNA-seq in CRISPR screen
Source: Genome Biol. 2020 Jun 8;21:136. doi: 10.1186/s13059-020-02044-w (PMC7278172; doi:10.1186/s13059-020-02044-w)
Supplement: Supplementary file 1 — Additional file 1: Figure S1. An illustration shows the stem extension when incorporating longer loop in order to main stable secondary structure. Figure S2. The off-target rate was examined for six known off-targeting sites of the VEGFA gRNA. Figure S3. Workflows of applying the programmed scaffold in different single cell RNA-seq platforms. Figure S4. The products from gRNA transcripts were effectively enriched by the nested PCR as exhibited from the fragment size analysis. Figure S5. The pearson correlation of UMI per cell between the two index gRNA libraries. Figure S6. Histogram of the gRNA UMI per cell from the index gRNA library II. Figure S7. Transcriptome profiling and KEGG enrichment of gRNA targeting genes in single cell sequencing. Figure S8. The sequencing reads from gRNA transcripts in each single cell were estimated using single cell RNA-seq conducted on the Fluidigm C1 platform. Figure S9. Different scaffold variants were examined, including G addition at different frequencies and A/G mixed sequence with different tractors. [file 13059_2020_2044_MOESM1_ESM.pdf]

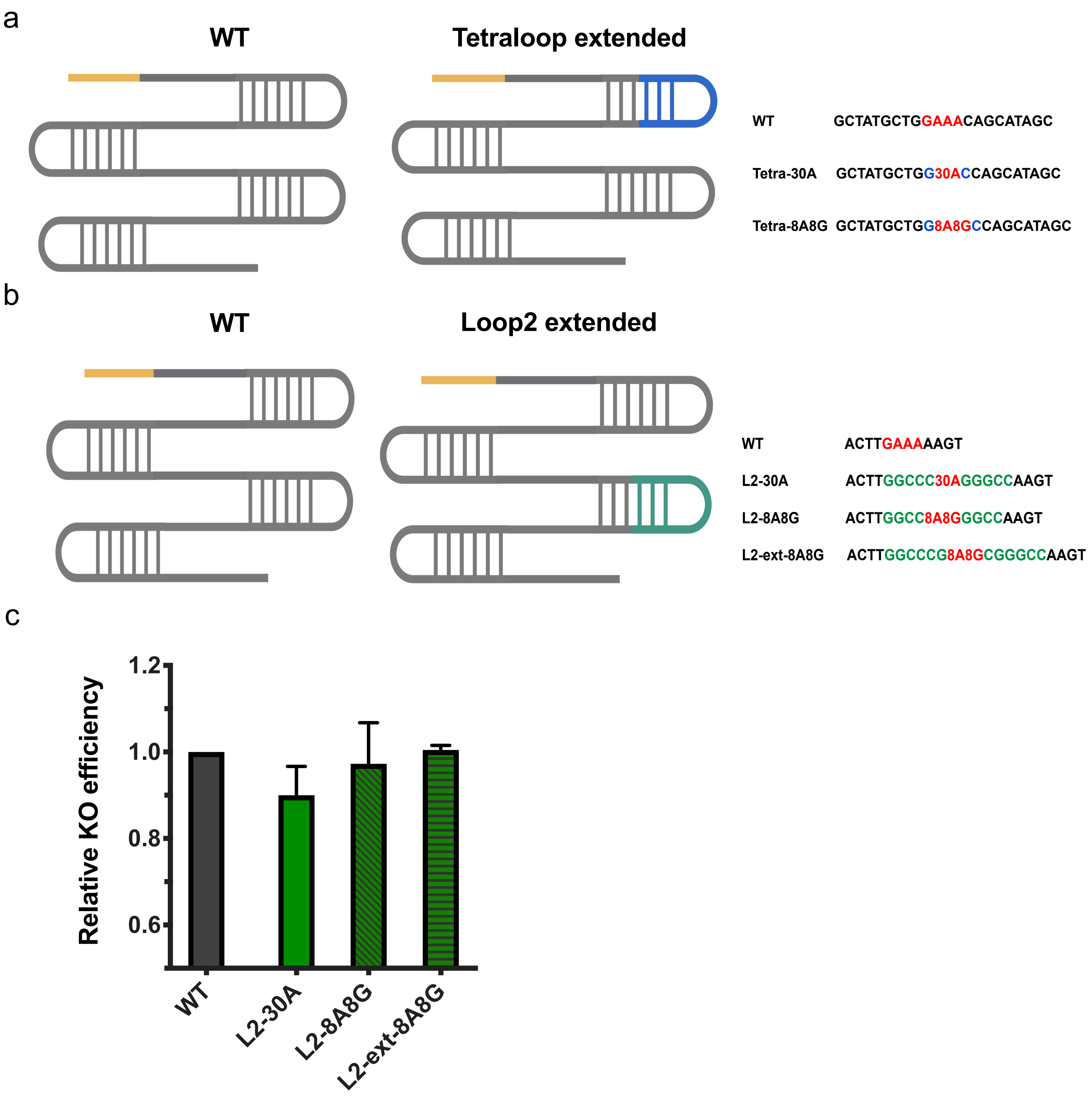

Fig. S1. An illustration shows the stem extension when incorporating longer loop in order to main stable secondary structure. (a) the extension at the stem region of Tetra loop. this extension was referred as Tetra-30A or Tetra-8A8G, and the efficiencies were evaluated in Fig1; (b) the extension at the stem region of the Loop2; (c) the editing efficiency increased when extended the stem region of Loop2.

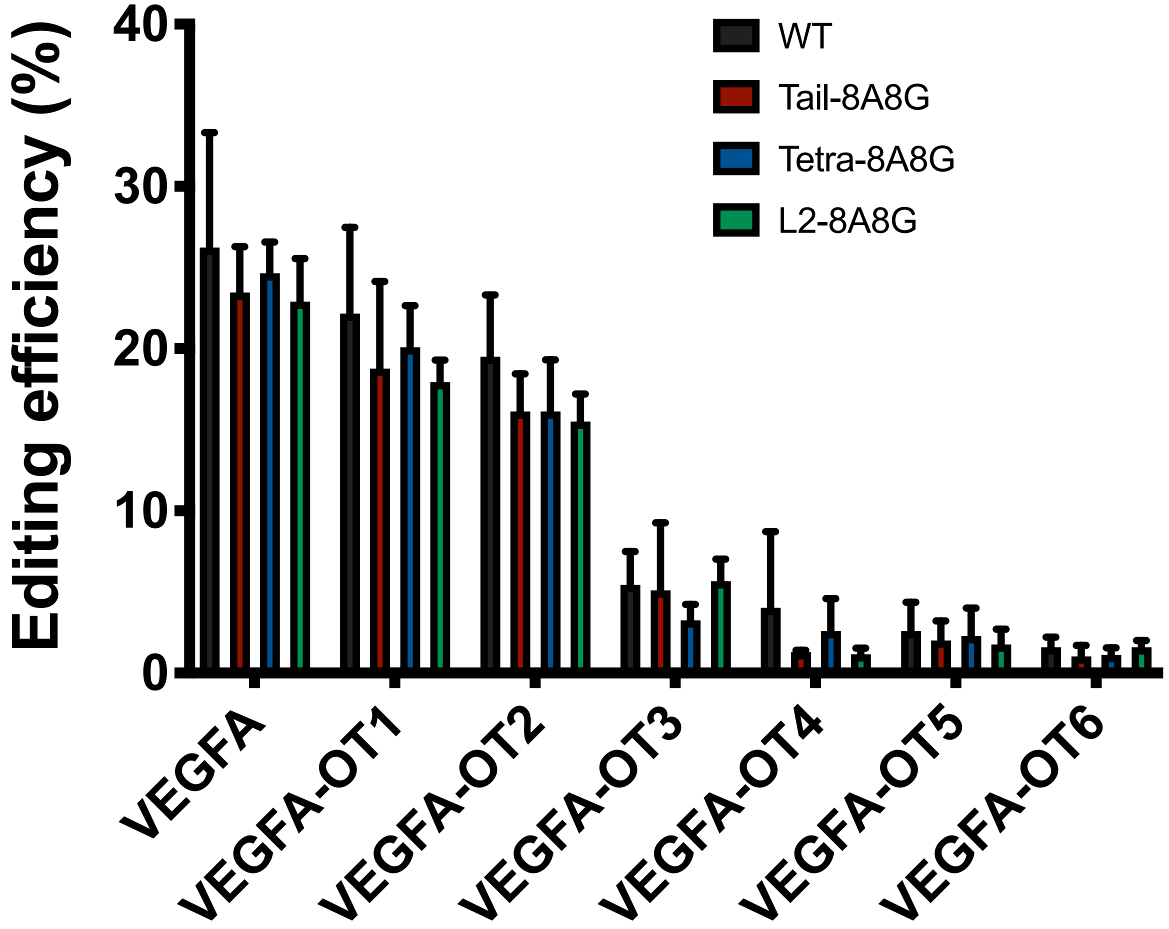

Fig. S2. The off-target rate was examined for six known off-targeting sites of the VEGFA gRNA. The programmed scaffolds show lower or similar off-targeting rates across all six sites we examined. This is a different VEGFA gRNA with known off-targeting sites, which we selected from previous publications.

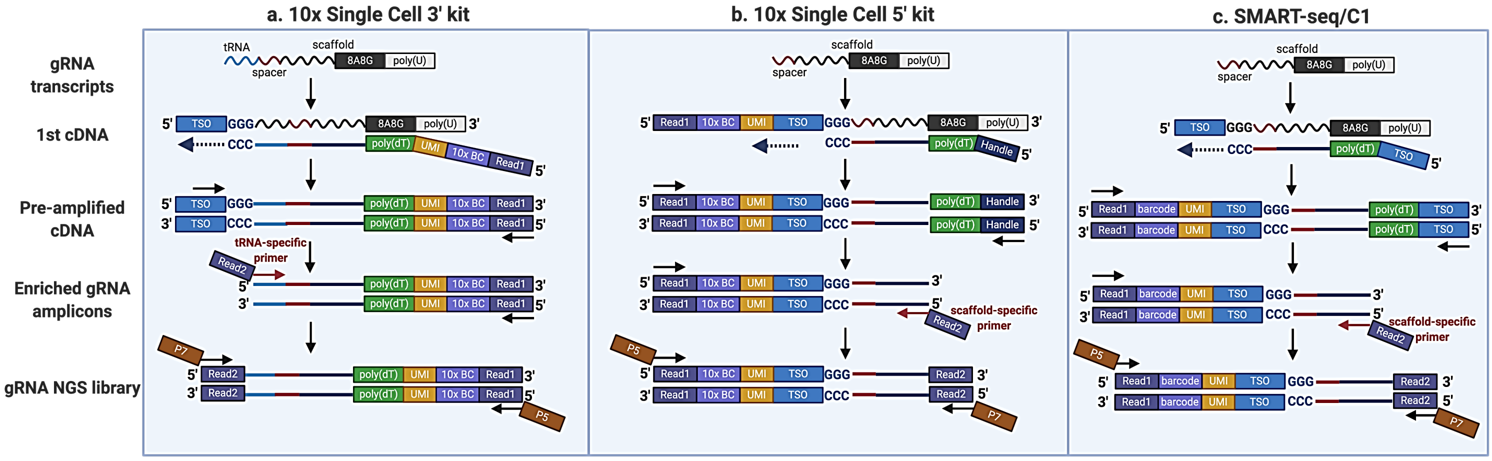

Fig. S3. Workflows of applying the programmed scaffold in different single cell RNA-seq platforms. (a) The suggested procedure to use with the 10x 3' kit; (b) The suggested procedure to use with the 10x 5' kit. The standard protocol should be followed until the pre-amplification of cDNA. The supernatant from size-selection contains gRNA sequences and could be specifically enriched by the nested PCR. The 1st step could use a scaffold-specific primer, and 2nd step could add the P5 and P7 sequencing adapters; (c) When work with the SMART-seq protocol, the cell barcode and UMI are suggested to be included in the template switch oligos (TSO) to enable sample pooling and eliminate PCR artifacts. The nested PCR is also suggested to make the gRNA library.

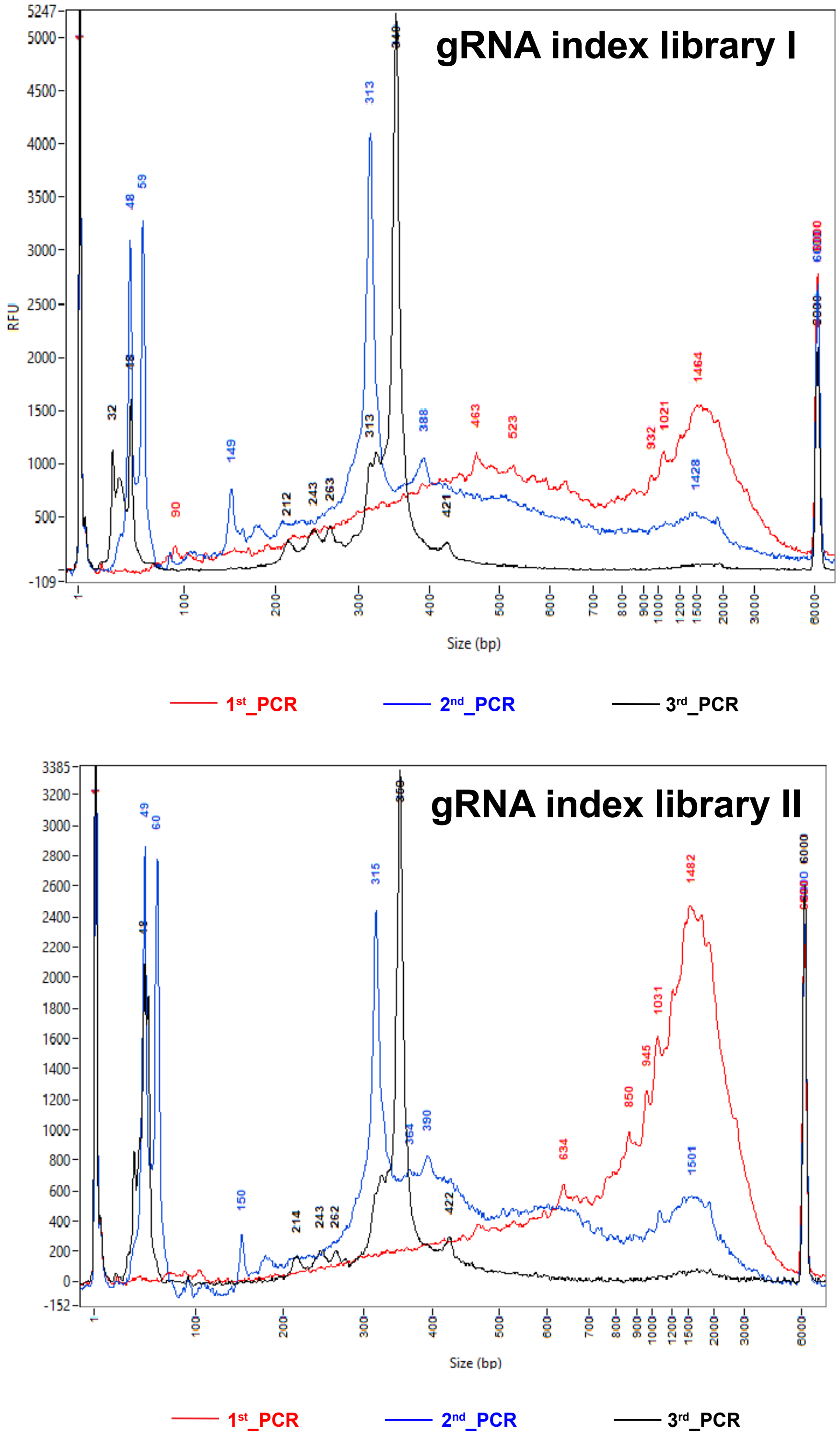

Fig. S4. The products from gRNA transcripts were effectively enriched by the nested PCR as exhibited from the fragment size analysis. 1st PCR: products from cDNA pre-amplification (expected products size: 294 bp); 2nd PCR: products from gRNA enrichment PCR (expected products size: 310 bp); 3rd PCR: products of the final index gRNA sequencing library (expected products size: 342 bp).

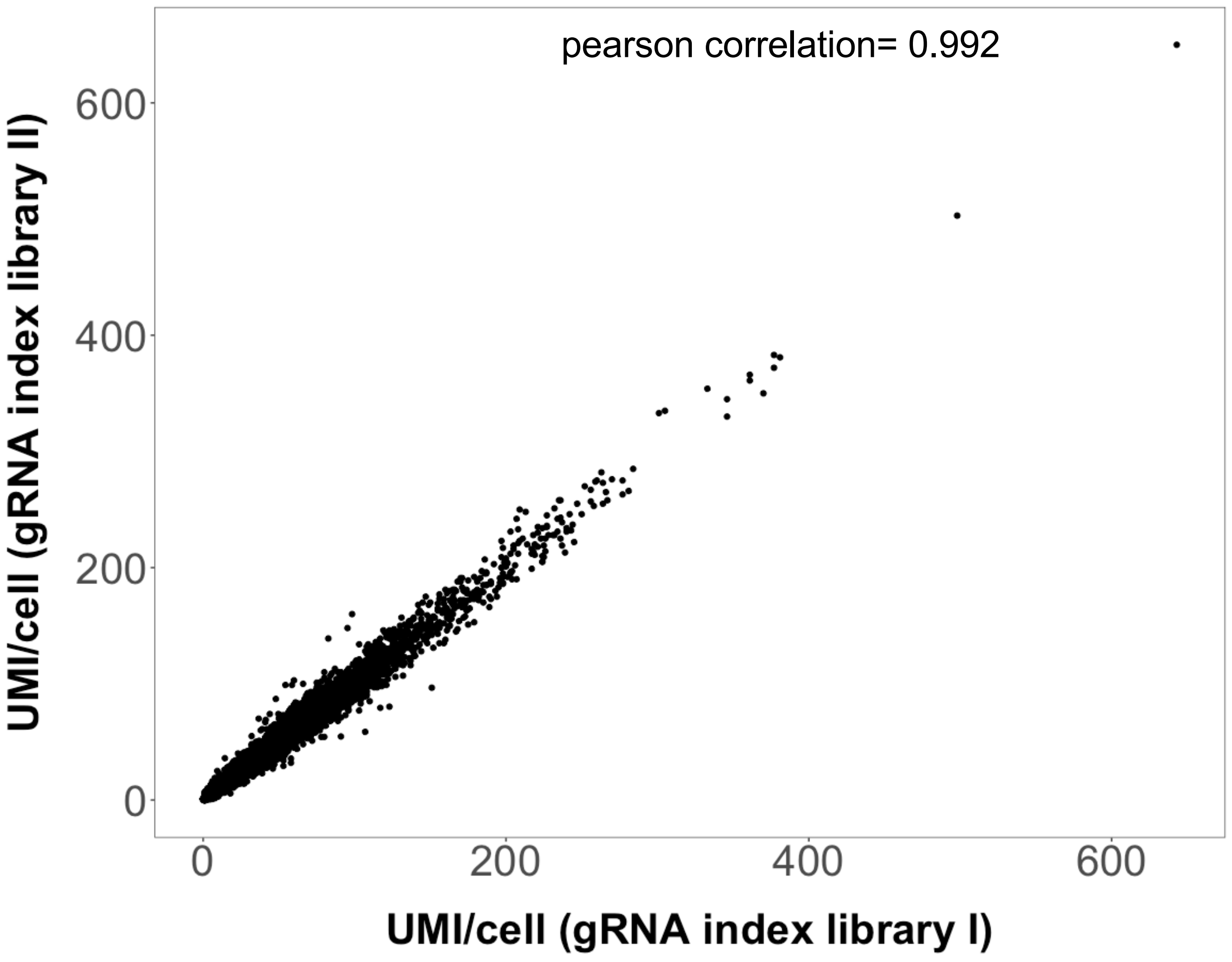

Fig. S5. The pearson correlation of UMI per cell between the two index gRNA libraries

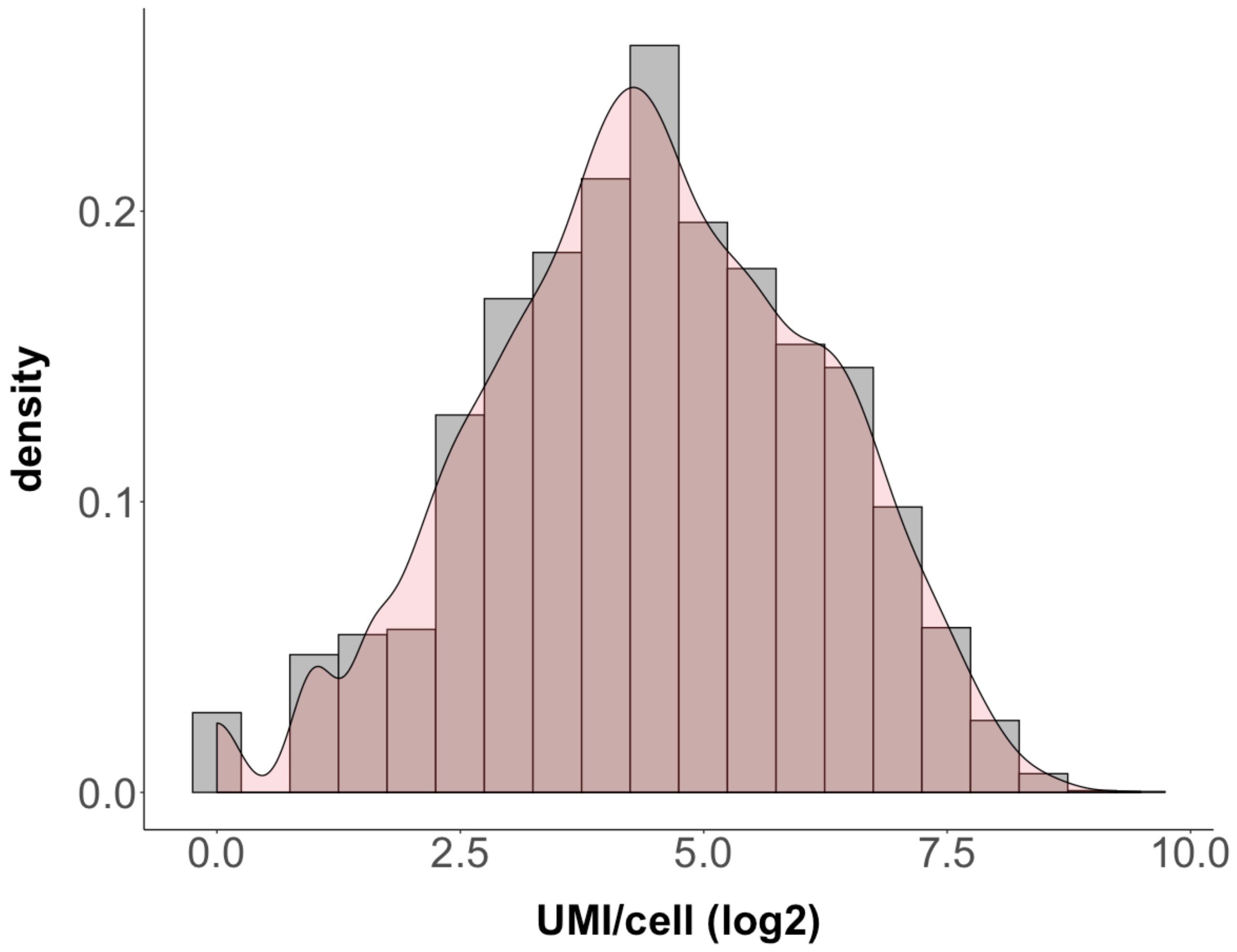

Fig. S6. Histogram of the gRNA UMI per cell from the index gRNA library II.

a

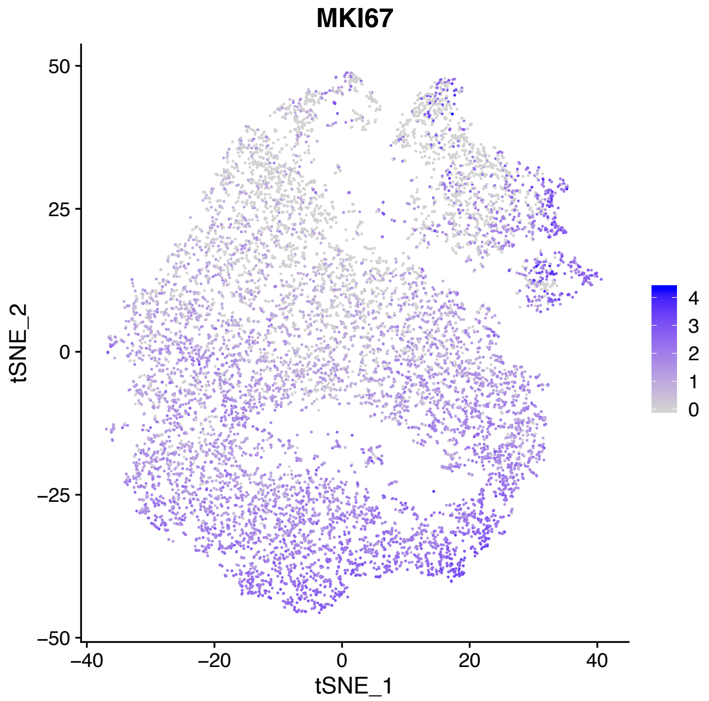

b

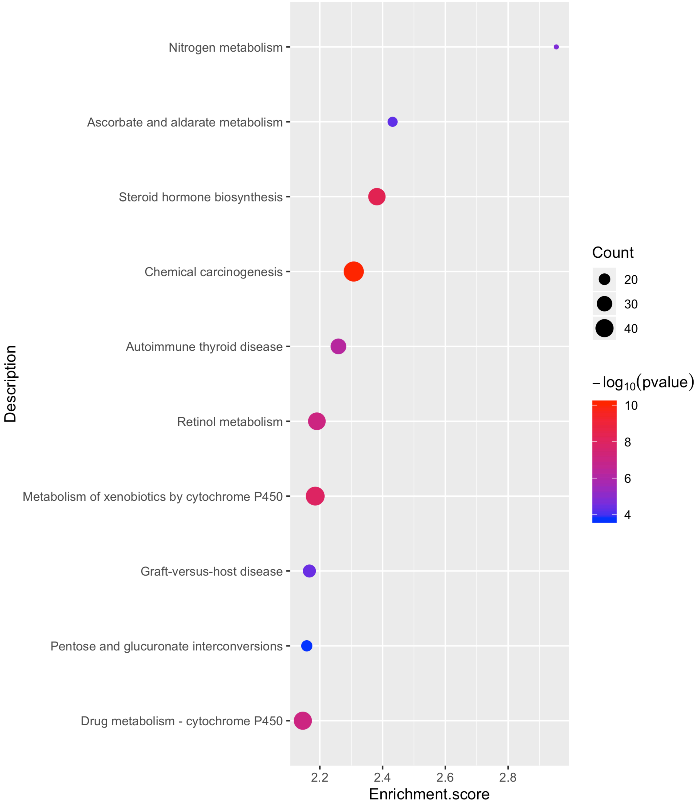

Fig. S7. Transcriptome profiling and KEGG enrichment of gRNA targeting genes in single cell sequencing. (a) Distribution of canonical proliferation marker genes MKI67 across clusters. (b) KEGG enrichment of gRNA targeting genes from cells highly expressed the marker genes.

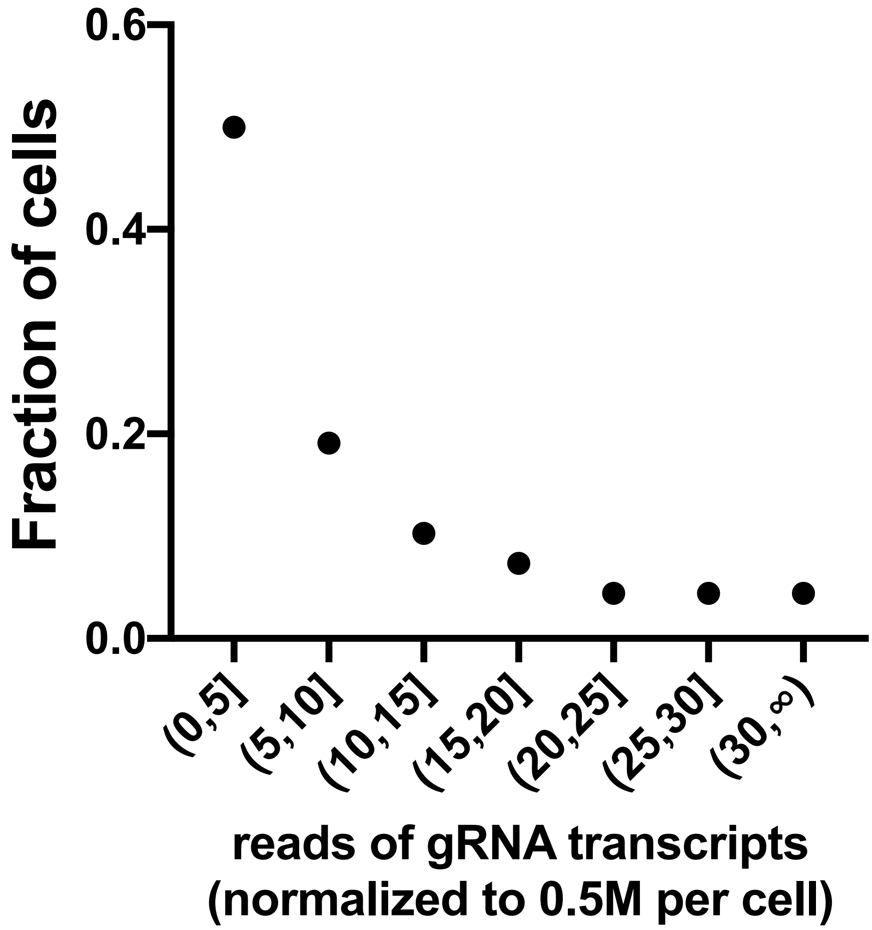

Fig. S8. The sequencing reads from gRNA transcripts in each single cell were estimated using single cell RNA-seq conducted on the Fluidigm C1 platform. In each cell, sequencing reads were normalized to 0.5 million, and more than 5 gRNA transcript reads can be detected in half of cells that we examined.

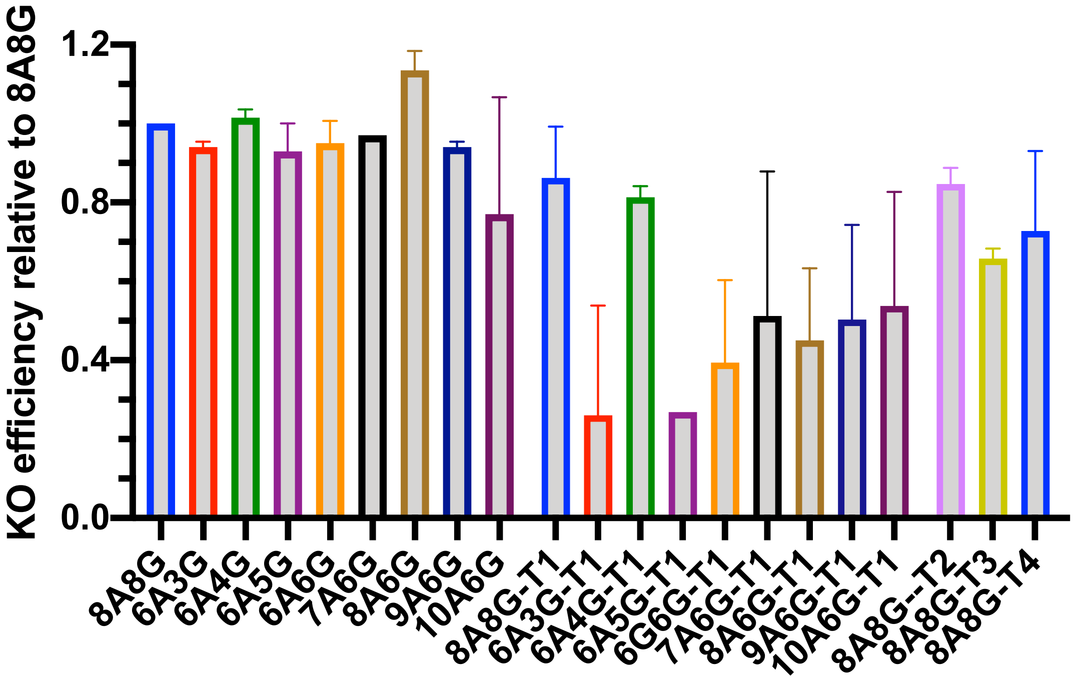

Fig. S9. Different scaffold variants were examined, including G addition at different frequencies and A/G mixed sequence with different tractors. All sequences can be found in Table S3.
