# Supplementary material for: Direct-seq: programmed gRNA scaffold for streamlined scRNA-seq in CRISPR screen
Source: Genome Biol. 2020 Jun 8;21:136. doi: 10.1186/s13059-020-02044-w (PMC7278172; doi:10.1186/s13059-020-02044-w)
Supplement: Supplementary file 6 — Additional file 6. Review history. [file 13059_2020_2044_MOESM6_ESM.docx]

Review History

**First round of review**

**Reviewer 1**

**Were you able to assess all statistics in the manuscript, including the appropriateness of statistical tests used?**

Yes, and I have assessed the statistics in my report.

**Comments to author:**

In this manuscript, the authors interrogate the idea of engineering the guide RNA scaffold to facilitate genotype-to-phenotype coupling in CRISPR screens. Overall the significance, rigor & extent of demonstration and novelty of the approach are low. Specifically, they explore adding a poly-A like capture sequence to facilitate direct capture of the gRNAs by poly-dT probes. In the course of this effort, the authors make two major claims.

The first major claim is that a poly-A like sequence, specifically the 8A8G construct can be added to the gRNA scaffold without a significant reduction in functionality of the Cas9-gRNA complex. The data generally support this claim, both in the CXCR4, VEGFA and DMD knockout experiments and the CRISPRa SAM gene activation, in which the data for the 8A8G gRNA construct recapitulate the findings with the wild-type gRNA (Figure 1a,b,d). However, I would recommend that the absolute knockout efficiency be presented along with the wild-type normalized data for clarity. Additionally, the x-axis intercept in Figure 1a should be at 0, or an axis break should be displayed so that a 24% decline in function (wild-type vs. Tail-30A) does not appear at first glance to be closer to a 50% reduction. Finally, an asterisk is displayed on some bars throughout the paper and denoted in the figure legend to indicate P < 0.05, however it is unclear what statistical procedure was used to generate this p-value.

One puzzling aspect of the data here is in Supplementary Figure 1 in which the authors test several poly-A like sequences comprised of poly-A stretches interspersed with G residues at periodic intervals. All of the other constructs aside from 8A8G (and perhaps 6A4G), show marked reduction in knockout efficiency, significantly more than any of the 30A constructs tested. It is unclear why this should be the case, and any potential reasons are not addressed by the authors. Though this does not necessarily affect the main findings, it is a strange and unexplained anomaly.

The second major claim is that the 8A8G sequence added at the tail region of the gRNA scaffold is sufficient to allow for capture using standard poly-dT protocols and subsequent genotyping of cells in CRISPR screens, particularly those with single-cell transcriptomic readouts. The authors are able to verify captured guides in cDNA from both bulk and single-cell RNA preparations at low quantities as expected. In addition, they were able to genotype a significant majority (68/73) of cells they analyzed in a single-cell screen (Figure 2d). However, the very low number of cells in this proof-of-concept screen is a poor comparison for a more typical single-cell transcriptomic use-case. Additionally, the low number of gRNA reads within the bulk transcriptome dataset would necessitate amplification in order to accurately genotype cells at a reasonable sequencing depth. The true sequencing depth in this experiment is unclear, as only the gRNA read count normalized to a very high depth (0.5M reads) is reported. The utility of this approach would be more convincing with a proof-of-principle experiment performed at a more usual scale with typical sequencing depth.

The final experiment in engineering the promoter element for the gRNA to increase transcript level is somewhat peripheral to the main point and is therefore less important, but its description creates some confusion. Firstly, it is not clear if what was added before the U6 promoter was the CMV promoter or the CMV enhancer (or both). Both terms are used in the main text, though they refer to different things. It is also not clear to me why the CMV promoter/enhancer and the human tRNA should increase knockout efficiency when the 8A8G construct is added at the tail region of the gRNA scaffold, but not in either of the other two loop regions tested.

Overall, the major claims are supported by the data, though the paradigm has not been fully tested at scale. The addition of poly-A type sequences to the guide RNA scaffold and capture of functional gRNAs directly is interesting and its suggested use to couple genotype to phenotype in single-cell screens (if fully demonstrated) is simpler and more accessible than barcode-based techniques or those based on non-poly-dT capture probes. However, it is unclear what improvement it offers over strategies such as CROP-seq in which the gRNA is also expressed via a polyadenylated Pol II transcript that can be captured with poly-dT probes. The problem to which this manuscript is directed, namely, that of coupling genotype to phenotype in CRISPR screens with single-cell transcriptomic readouts, has several potential solutions. This work represents a new and distinct, though not obviously better way to solve it. The authors should expand on what kinds of problems this technique is uniquely placed to address and why. Finally, the manuscript contains many grammatical, spelling, and idiomatic errors, and needs to be heavily copy-edited.

**Reviewer 2**

**Were you able to assess all statistics in the manuscript, including the appropriateness of statistical tests used?**

No, I do not feel adequately qualified to assess the statistics.

**Comments to author:**

In this manuscript, Song et al. present a flexible and cost-effective strategy to simultaneously measure guide RNA expression with transcriptome responses in single cells. Previous methods (Perturb-seq, CRISP-seq and CROP-seq) link the genotype, transcriptome and phenotypes through poly-adenylated gRNA copy or gRNA barcode, which involve complicated cloning strategy and sometime rely on the decoupling of spacer sequence and its barcode (Dixit et al., Cell, 2016; Jaitin et al., Cell, 2016; Datlinger et al., Nat methods, 2017). This study demonstrates that the gRNA transcripts can be captured directly by oligo(dT), together with the endogenous mRNA, through introducing an A/G mixed sequence to the scaffold RNA. Hence, this method is much easier and cost-effective. I would recommend the publication of this study given my concerns below can be satisfyingly addressed.
Major Points:
1. A previous study demonstrated that RNA secondary structure is a fundamental parameter that can tune the nuclease activity and specificity of diverse CRISPR systems (D. Dewran Kocak et al., Nat Biotechnol, 2019). While the authors show that a mixed adenosine/guanine sequence does not cause negative impact to the on-target efficiency of the CRISPR/Cas9 and CRISPRa systems, the authors did not assess the off-target risk due to a mixed adenosine/guanine sequence. I recommend the authors chose spacers that have large numbers of well-characterized off-target sites (Tsai et al., Nat Biotechnol, 2015) to assess whether inserting a mixed adenosine/guanine sequence at different locations increases the risk of off-targeting.
2. Fig 1c: What's the capture efficiency of WT gRNA relative to "30A"? I suggest to take the capture efficiency of WT gRNA relative to "30A" as a negative control in this experiment, which can strongly prove the result from Fig 1c.
3. Fig 2c: Media only was used as a negative control in this experiment, which may be unreasonable. Also, the Ct value of media only is too high as negative controls. Hence, I suggest the authors use the individual cells transfected wide type gRNA as negative controls.
4. Fig 2b-d: Bulk cell RNA-seq data demonstrating that the expression level of the HBG1 and its homology HBG2 (also the target of the same spacer) were significantly unregulated resulting from the HBG1-8A8G perturbation as expected (Lines 377-381).  It is particularly important to show the same result at the single cell level in the cells isolated from the HBG1-8A8G perturbation.
Minor Points:
1. Fig1a: The text describing the editing efficiency should be corrected as follows, "The results showed that the editing efficiency dropped 24% with the 30A insertion at the Tail position, whereas 13% and 12% with the insertion at the Tetraloop and Loop2 respectively." (lines 70-72)
2. sFig2: The authors state that "…extending the stem length of both Tetraloop and Loop2 can further increase the editing efficiency slightly." (Line 96) As shown in sFig3, there exists no obvious difference in KO efficiency between L2-8A8G and L2-ext1-8A8G. Furthermore, data of extending the stem length of Tetraloop should be provided.
3. Fig1b: In this expriment, which of the three locations (Tetra, Loop2 and Tail) that the 8A8G sequence inserted in? And whether extend the stem or not ?This should be described in figure legend or results.

**Authors Response**

**Point-by-point responses to the reviewers’ comments:**

Reviewer #1: In this manuscript, the authors interrogate the idea of engineering the guide RNA scaffold to facilitate genotype-to-phenotype coupling in CRISPR screens. Overall the significance, rigor & extent of demonstration and novelty of the approach are low. Specifically, they explore adding a poly-A like capture sequence to facilitate direct capture of the gRNAs by poly-dT probes. In the course of this effort, the authors make two major claims.
*Thanks for your time! We have redesigned the demonstration case, reorganized the contents and rewrote many paragraphs of the manuscript. We hope that the revised manuscript could be more convincing to demonstrate the methodology advantage of using the programmed scaffold in single cell CRISPR screen.*

The first major claim is that a poly-A like sequence, specifically the 8A8G construct can be added to the gRNA scaffold without a significant reduction in functionality of the Cas9-gRNA complex. The data generally support this claim, both in the CXCR4, VEGFA and DMD knockout experiments and the CRISPRa SAM gene activation, in which the data for the 8A8G gRNA construct recapitulate the findings with the wild-type gRNA (Figure 1a,b,d). However, I would recommend that the absolute knockout efficiency be presented along with the wild-type normalized data for clarity. Additionally, the x-axis intercept in Figure 1a should be at 0, or an axis break should be displayed so that a 24% decline in function (wild-type vs. Tail-30A) does not appear at first glance to be closer to a 50% reduction. Finally, an asterisk is displayed on some bars throughout the paper and denoted in the figure legend to indicate P < 0.05, however it is unclear what statistical procedure was used to generate this p-value.
*A: That are all very good suggestions, thank you!
(1) The absolute knockout efficiencies have been included and summarized in Supplementary Table1.
(2) The y-axis of Fig1a has been edited as suggested.
(3) The “asterisk” has been explained in the figure legends for clarification.*

One puzzling aspect of the data here is in Supplementary Figure 1 in which the authors test several poly-A like sequences comprised of poly-A stretches interspersed with G residues at periodic intervals. All of the other constructs aside from 8A8G (and perhaps 6A4G), show marked reduction in knockout efficiency, significantly more than any of the 30A constructs tested. It is unclear why this should be the case, and any potential reasons are not addressed by the authors. Though this does not necessarily affect the main findings, it is a strange and unexplained anomaly.
*A: Thanks for pointing this out. We combined the data of the previous Supplementary Figure 1 and Supplementary Figure 4 together in the revised manuscript as Supplementary Figure 9, which served as a discussion on the editing efficiencies of different capture sequences under the same rationale. As the reviewer has pointed out, we considered that the major point of this manuscript was that a programmed scaffold with an A/G mixed capture sequence could be reverse transcribed efficiently by poly(dT) and serve as index gRNA using a very straightforward workflow. Although we demonstrated the performance of the 8A8G in multiple CRISPR perturbations and on different scRNA-seq platforms, we cannot rule out that other A/G mixed sequences may work in similar pattern, as it’s difficult to exhaust all possible combinations. Therefore, we decided to move this plot from “Results” to “Discussion” for future follow-up.*

The second major claim is that the 8A8G sequence added at the tail region of the gRNA scaffold is sufficient to allow for capture using standard poly-dT protocols and subsequent genotyping of cells in CRISPR screens, particularly those with single-cell transcriptomic readouts. The authors are able to verify captured guides in cDNA from both bulk and single-cell RNA preparations at low quantities as expected. In addition, they were able to genotype a significant majority (68/73) of cells they analyzed in a single-cell screen (Figure 2d). However, the very low number of cells in this proof-of-concept screen is a poor comparison for a more typical single-cell transcriptomic use-case. Additionally, the low number of gRNA reads within the bulk transcriptome dataset would necessitate amplification in order to accurately genotype cells at a reasonable sequencing depth. The true sequencing depth in this experiment is unclear, as only the gRNA read count normalized to a very high depth (0.5M reads) is reported. The utility of this approach would be more convincing with a proof-of-principle experiment performed at a more usual scale with typical sequencing depth.
*A: Thanks for the constructive suggestion!
To demonstrate the application of the programmed scaffold in a more typical scale, we performed a single cell RNA-seq experiment using the 10x Single Cell 3’ Library Kit v3 (#PN-1000075), in order to provide a more comprehensive assessment on the capturability of index gRNAs with typical sequencing depth. As we have summarized in the new Fig2 and Supplementary Table4, 99.4% cell barcodes from the mRNA library were recovered from the index gRNA libraries, at the sequencing depths of ~2,000 reads per cell. This identification rate is even higher than a recently published Nature Biotech paper, in which they used the CS1 and CS2 capture sequences come with the 10x 3’ Single Cell v3 kit (84~94%, Fig1d from J Replogle et al, Nature Biotech 2020). We carefully compared our library preparation procedure with theirs, and thought that the nested PCR we employed to generate the index gRNA libraries helped to better enrich sequences containing the gRNAs (Supplementary Figure4 of the revised manuscript, Supplementary Note 3 from J Replogle et al, Nature Biotech 2020). The 1st step of the nested PCR used a gRNA specific primer, which may eliminate the background of non-specific cDNA products.
Accordingly, in the previous demonstration using the C1 platform, which employed the Takara SMART-seq v4 protocol, we did not apply the nested PCR. The required sequencing depth to identify gRNAs out of the transcriptome was very high. Therefore, we made suggestions in the Supplementary Figure3c, which shows how to apply the nested PCR with the SMART-seq protocol.*

The final experiment in engineering the promoter element for the gRNA to increase transcript level is somewhat peripheral to the main point and is therefore less important, but its description creates some confusion. Firstly, it is not clear if what was added before the U6 promoter was the CMV promoter or the CMV enhancer (or both). Both terms are used in the main text, though they refer to different things. It is also not clear to me why the CMV promoter/enhancer and the human tRNA should increase knockout efficiency when the 8A8G construct is added at the tail region of the gRNA scaffold, but not in either of the other two loop regions tested.
*A: Thanks for pointing this out! In the previous Fig2e-f, the “CMV” actually referred to a 378bp “CMV enhancer”, which also includes some promoter elements. We removed the “CMV enhancer” results as it was loosely connected to the main point, just as the reviewer has pointed out.
We retained the tRNA results and moved the plot as Figure2a in the revised manuscript. In order to use the programmed scaffold in the 10x 3’ single cell kit, we incorporated a tRNA sequence into the gRNA expression cassette. The tRNA, which located in-between the U6 promoter and gRNA, provided a specific primer binding site for the nested PCR. Many previous studies have used the tRNA together with gRNA in order to either boost gRNA expression level, or process multiple gRNAs from the same transcript (Mefferd et al. RNA 2015; Xie et al. PNAS 2015, Knapp et al. Nat. Commun. 2019 and Zhang et al. Nat. Commun. 2019). Our data also indicated that the incorporation of human Gln tRNA won’t influence the editing efficiency. As this part of results now supported the utility of tRNA in the following 10x single cell experiment, we kept it as the first panel of Figure2.*

Overall, the major claims are supported by the data, though the paradigm has not been fully tested at scale. The addition of poly-A type sequences to the guide RNA scaffold and capture of functional gRNAs directly is interesting and its suggested use to couple genotype to phenotype in single-cell screens (if fully demonstrated) is simpler and more accessible than barcode-based techniques or those based on non-poly-dT capture probes. However, it is unclear what improvement it offers over strategies such as CROP-seq in which the gRNA is also expressed via a polyadenylated Pol II transcript that can be captured with poly-dT probes. The problem to which this manuscript is directed, namely, that of coupling genotype to phenotype in CRISPR screens with single-cell transcriptomic readouts, has several potential solutions. This work represents a new and distinct, though not obviously better way to solve it. The authors should expand on what kinds of problems this technique is uniquely placed to address and why. Finally, the manuscript contains many grammatical, spelling, and idiomatic errors, and needs to be heavily copy-edited.
*A: Thanks for the comments, we realized that the advantages of the programmed scaffold haven’t been discussed sufficiently in the previous version of manuscript. Therefore, we rewrote the entire demonstration section and discussion section, and polished most of other parts of the manuscript. In the revised manuscript, we emphasized the streamlined workflow, high gRNA identification rate, compatibility with combinatorial perturbation (to CROP-seq), and flexibility to adapt on versatile scRNA-seq platforms.
The language has also been polished and we apologize if it hasn’t been completely addressed. But we’re very happy to send the manuscript for professional language support after the revision.*
 
Reviewer #2: In this manuscript, Song et al. present a flexible and cost-effective strategy to simultaneously measure guide RNA expression with transcriptome responses in single cells. Previous methods (Perturb-seq, CRISP-seq and CROP- seq) link the genotype, transcriptome and phenotypes through poly-adenylated gRNA copy or gRNA barcode, which involve complicated cloning strategy and sometime rely on the decoupling of spacer sequence and its barcode (Dixit et al., Cell, 2016; Jaitin et al., Cell, 2016; Datlinger et al., Nat methods, 2017). This study demonstrates that the gRNA transcripts can be captured directly by oligo(dT), together with the endogenous mRNA, through introducing an A/G mixed sequence to the scaffold RNA. Hence, this method is much easier and cost-effective. I would recommend the publication of this study given my concerns below can be satisfyingly addressed.
*A: Thank you very much for the positive feedback and constructive suggestions! As you will see below, we have answered all the concerns point-by-point. Additionally, per the requested from another review and the editor, we have conducted a single cell CRISPR screen and characterized cells using the 10x platform to demonstrate the performance of our programmed scaffold. Based on the data, we summarized the advantages of the new methodology over the other methods in the “Discussion” section, including streamlined workflow, high gRNA identification rate, compatibility with combinatorial perturbation, and flexibility to adapt on versatile scRNA-seq platforms.*

Major Points:
1. A previous study demonstrated that RNA secondary structure is a fundamental parameter that can tune the nuclease activity and specificity of diverse CRISPR systems (D. Dewran Kocak et al., Nat Biotechnol, 2019). While the authors show that a mixed adenosine/guanine sequence does not cause negative impact to the on-target efficiency of the CRISPR/Cas9 and CRISPRa systems, the authors did not assess the off-target risk due to a mixed adenosine/guanine sequence. I recommend the authors chose spacers that have large numbers of well-characterized off-target sites (Tsai et al., Nat Biotechnol, 2015) to assess whether inserting a mixed adenosine/guanine sequence at different locations increases the risk of off-targeting.
*A: Thanks for this very constructive suggestion! This is an important piece that should be included. We have referred to publications and examined six known off-targeting sites of VEGFA gRNA. The results indicated there’s no influence on the overall off-targeting rates across all sites we’ve examined. The results have been summarized in the new Supplementary Figure2.*
2. Fig 1c: What's the capture efficiency of WT gRNA relative to "30A"? I suggest to take the capture efficiency of WT gRNA relative to "30A" as a negative control in this experiment, which can strongly prove the result from Fig 1c.
*A: Thanks for the suggestion! We have included the WT signal to the Figure1c, which should help to reveal the capture efficiency of different scaffolds compared to the wild type.*

3. Fig 2c: Media only was used as a negative control in this experiment, which may be unreasonable. Also, the Ct value of media only is too high as negative controls. Hence, I suggest the authors use the individual cells transfected wide type gRNA as negative controls.
*A: Thanks for the comments! The high Ct value is an issue in this type of experiment due to ambient transcripts. Besides, another review also raised concern that these experiments can’t fully support the capturability of the programmed scaffold. Therefore, we redesigned the demonstration case, and performed a single cell CRISPR screen and characterized the index gRNA using the 10x 3’ single cell RNA-seq kit. This part of results has been replaced by the new Figure2.*

4. Fig 2b-d: Bulk cell RNA-seq data demonstrating that the expression level of the HBG1 and its homology HBG2 (also the target of the same spacer) were significantly unregulated resulting from the HBG1-8A8G perturbation as expected (Lines 377-381). It is particularly important to show the same result at the single cell level in the cells isolated from the HBG1- 8A8G perturbation.
*A: Thanks for pointing this out. As mentioned above, the entire panel of the previous Figure2 has been replaced by the 10x data, except the previous Figure2d, which now has been moved to Supplementary Figure8.
However, to answer the question, we checked our record of the bulk cell (previous Figure2a-b) and single cell RNA-seq data (previous Figure2c) relative to the HBG1/2 CRISPR activation experiments. From the scRNA-seq data of two individual single cells (as biological replicates), the expression level of HBG1 and HBG2 increased from 0 to 18.64 and 9.88 (TPM, transcripts per million), with log2FC 3.93. Although the analysis of these data has been removed, the raw data will be uploaded to the GEO database together with the 10x and C1 scRNA-seq data.*

Minor Points:
1. Fig1a: The text describing the editing efficiency should be corrected as follows, "The results showed that the editing efficiency dropped 24% with the 30A insertion at the Tail position, whereas 13% and 12% with the insertion at the Tetraloop and Loop2 respectively." (lines 70-72)
*A: The text has been changed as suggested. Thank you!*

2. sFig2: The authors state that "...extending the stem length of both Tetraloop and Loop2 can further increase the editing efficiency slightly." (Line 96) As shown in sFig3, there exists no obvious difference in KO efficiency between L2-8A8G and L2-ext1-8A8G. Furthermore, data of extending the stem length of Tetraloop should be provided.
*A: Thank you for pointing this out. We have removed the L2-ext1-8A8G and kept the L2-ext2-8A8G (renamed as L2-ext-8A8G) to avoid confusion.
The extension to the stem region of the Tetraloop has been employed in the “Tetraloop-30A” and “Tetraloop-8A8G” scaffold as shown in the previous Supplementary Figure2a. And the editing efficiency of the Tetraloop-30A and the Tetraloop-8A8G were presented in the previous Figure1a (as well as the new Figure1a). However, we changed the color scheme of the illustration to avoid confusion.*

3. Fig1b: In this expriment, which of the three locations (Tetra, Loop2 and Tail) that the 8A8G sequence inserted in? And whether extend the stem or not? This should be described in figure legend or results.
*A: Thank you for pointing this out. We have edited the figure legend and main text to clarify. The 8A8G sequence was inserted in the Tail position in this experiment, without extension.*

**Second round of review**

**Reviewer 1**

On the technical front, the new experiment using the 10X platform seems to have addressed the critical scale issue, and the new PCR technique they use to amplify the gRNAs is more solid.

Important though is the question of what advantages the method offers over existing approaches such as CROP-Seq or the commercial 10X method. The arguments about simplicity and multiplexability are still underdeveloped and not very convincing. The authors must discuss this more thoroughly.

**Reviewer 2**

In this manuscript, Song et al. present a method that the CRISPR perturbation and its transcriptional readouts can be profiled together in a streamlined workflow by introducing an A/G mixed capture sequence into the gRNA scaffold. I think the author satisfactorily responded to my previous review.

**Authors Response**

**Point-by-point responses to the reviewers’ comments:**

Comment

*Response*
